# Cytokines Modulate the “Immune-Metabolism” Interactions during Behçet Disease: Effect on Arginine Metabolism

**DOI:** 10.1155/2015/241738

**Published:** 2015-01-27

**Authors:** Houda Belguendouz, Karima Lahmar-Belguendouz, Djamel Messaoudene, Zineb Djeraba, Fifi Otmani, Djennat Hakem, Ouided S. Lahlou-Boukoffa, Pierre Youinou, Chafia Touil-Boukoffa

**Affiliations:** ^1^USTHB, “Cytokines and NO Synthases” Team, LBCM, FSB, BP 32 El Alia, Bab Ezzouar, 16111 Algiers, Algeria; ^2^Internal Medicine Department, CHU Mustapha Bacha, Algiers, Algeria; ^3^Internal Medicine Department, CHU Bab El Oued, Algiers, Algeria; ^4^Ophthalmology Department, CHU Ibn Rochd, Annaba, Algeria; ^5^EA 2216, UBO, Brest, France

## Abstract

*Aim and Methods*. In this study, we evaluated NOS and arginase activities and their regulation during Behçet disease, a systemic chronic inflammatory disorder with uncertain etiology. The peripheral blood mononuclear cells of 36 patients and 15 control samples (PBMC) were cultured in either RPMI 1640, MEM, or DMEM complemented with 10% of FBS and antibiotics. Cultures were performed with or without the control or patients plasma. Subsequent treatment contained anticytokines (IL-6, TGF-*β*), a mitogenic effector (PHA), or NOS modulators (L-NMMA, BH4). Culture supernatants were harvested after 24 h of incubation. NO and urea measurements were, respectively, performed by modified Griess and Berthelot methods. *Results*. Higher urea levels were found in patients' plasma compared to the control's (*P* < 0.05). NOS modulators induced inverted production profiles for NO and urea (*P* < 0.05). Their results differed depending on the clinical findings (*P* < 0.05). It was also found that cytokine neutralization induced different response profiles in patients as opposed to control cultures (*P* < 0.05). *Conclusion*. Our results suggest that arginases can compete with NOS2 for L-arginine during Behçet disease. Both enzymes are regulated by environmental cytokines and substrate availability. Furthermore, it seems that NOS/arginase balance is dependent on clinical expression.

## 1. Introduction

Behçet's disease (BD) is a chronic systemic inflammatory disorder characterized by a course of remissions and exacerbations of unpredictable frequency and duration. This disease is considered as vasculitis, affecting vessels of different types, sizes, and localizations [1]. It is most prevalent in countries of the Mediterranean area, including Algeria, and along the Silk Route. The clinical features include oral and genital ulcers, ocular and skin lesions, and central nervous system, joint, vascular, gastrointestinal, or pulmonary manifestations. The treatment comprised systemic corticosteroids and immunosuppressants [2].

Despite the presence of regional and ethnical variability in the clinical expression, uveitis is one of the major disease manifestations [3]. Although it is not a life threatening manifestation, it has a heavy socioeconomic impact. In fact, blindness frequency, due to relapsing ocular inflammation, occurs in about 70% of the patients, even when intensive immunosuppressive therapy is provided [4].

The cause and pathogenesis of BD are still unknown, but the onset of the disease is believed to be triggered by external environmental factors (Herpes simplex virus and streptococcal infection) in subjects with particular genetic susceptibilities (association with HLA-B51, MICA genes). Immunoregulatory abnormalities suggesting an autoimmune context have also been proposed as pathogenic mechanisms [5]. Moreover, several reports suggested that autoimmunity mechanisms may play a crucial role. The presence of immunoreactivity against vascular and ocular structures especially retinal proteins such as visual arrestin (S-Ag) and interphotoreceptor retinoid-binding protein (IRBP) had been reported [6, 7]. Furthermore, some clinical and pathological findings suggest an autoinflammatory component [8]. Indeed, neutrophil hyperactivity has also been suggested as one of the main pathogenic mechanisms in BD [9]. In addition, overexpression of pattern recognition receptors (PRRs) as TLR2 and TLR 6 has been shown on patient's immune cells [10, 11].

Cytokines play a key role in Behçet physiopathology. In the last few years, an increasing amount of data has reported the involvement of chemokines (IL-8, MPC-1), proinflammatory cytokines (IL-1*β*, TNF-*α*), and immune-modulatory cytokines (Th differentiation driven markers) in disease pathogenicity and/or activity [12]. In the same way, the production of tumor necrosis factor (TNF-*α*), IL-6, and IL-8 has been found to be elevated [13]. Furthermore, spontaneous or induced overexpression of proinflammatory and Th1 type cytokines has been shown in various cellular sources. Proinflammatory cytokines seem to be responsible for the enhanced inflammatory response in BD [14, 15]. In our previous studies, we reported the overproduction of either Th1 (IL-12, IFN-*γ*), Th2 (IL-4), or Treg (IL-10) cytokines during the different stages of the disease [16–19]. In addition, we observed an increase in inflammatory markers production (IL-8 and nitric oxide) during Behçet uveitis. Furthermore, the elevation of IL-8 and nitric oxide was correlated with disease activity. Interestingly, patients' treatment with corticoids during active stages resulted in a significant diminution in the production of these molecules in relation to the clinical stages of the disease [20].

Nitric oxide (NO) is coproduced with L-citrulline by NO synthase (NOS) from L-arginine [21]. In return, this semiessential amino acid can be regenerated from L-citrulline by the sequential action of the cytosolic enzymes argininosuccinate synthetase (ASS) and argininosuccinate lyase (ASL) forming the nitric oxide cycle [22]. L-Arginine can also be transformed to L-ornithine and urea through the action of arginases (ARG) establishing a competition between NOS and ARG for their common substrate [23]. However, as L-ornithine can lead to the synthesis of L-citrulline and, thereby, to the synthesis of L-arginine via the urea cycle, the relationship between the two enzymes families depends on arginine availability and the presence of urea cycle enzymes [24].

In our study, we studied the activity of NOS and ARG during Behçet disease* in vivo*. Furthermore, we searched the presence of NO cycle in Behçet disease and the* ex vivo* effects of cytokines on enzyme activity.

## 2. Patients and Methods

### 2.1. Patients and Samples

We included 36 Algerian patients (21 men and 15 women) fulfilling the diagnostic criteria of the International Study Group for BD. Patients were followed in ophthalmology and internal medicine departments of Mustapha Bacha, Ibn Rochd, and Bab El Oued CHU. The mean (SD) duration of the disease was 9.5 ± 5.7 years (range 1.5–21), and the patients ages ranged from 21 to 58 years (mean 26). Patients' clinical characteristics are summarized in Table 1. All patients were treated with low doses of corticoids and colchicine. Patients with arthritis were receiving aspirin at low doses. We excluded all patients under immunosuppressant therapy. Sex- and age-matched healthy volunteers were included as normal controls (*n* = 23). All subjects in this study provided informed consent and the study was approved by the local Ethics Committee (ATRSS: Algerian national agency for research in health sciences).

### 2.2. Materials

All chemicals were of the highest analytical grade and were purchased from Sigma-Aldrich unless it is cited in the text.

### 2.3. Cells Separation and Culture

Peripheral blood mononuclear cells (PBMCs) were obtained by separating heparinized venous blood on Histopaque as previously described [20]. Viable mononuclear cells that excluded Trypan blue were counted (viability always >98%) and then diluted in either RPMI medium with FBS at 5%, MEM, or DMEM with FBS at 10% to a concentration of 10^6^ cells/mL. All experiments were performed in multiwell cell culture plates, and cultures were set up in triplicate. Cultures of PBMCs were stimulated by the addition of either patient's or control's plasma (20%) with or without phytohaemagglutinin (PHA, 10 *μ*g/mL), L-arginine (1 mM), BH4 (10 *μ*M), L-NMMA (10 ng/mL), or neutralizing anticytokines (anti-IL-6, anti-TGF-*β*) at 20 *μ*g/mL. Neutralizing human IgG antibody was used as an antibody control at 20 *μ*g/mL. Cultures were incubated for 24 h in 5% CO_2_ at 37°C in humidified chamber. After incubation, supernatants were stored at −70°C.

### 2.4. Cytokine Measurements

Commercial ELISA sandwich kits were used to measure plasmatic IL-6 (Immunotech, Beckman Coulter, France) and TGF-*β* (Life Technologies, CA). The lowest level of sensitivity was 6 pg/mL for IL-6 and 25 pg/mL for TGF-*β*.

### 2.5. Urea Measurement

Urea was measured by Berthelot method. In brief, the enzyme urease is used to catalyze the hydrolysis of urea into carbon dioxide and ammonia. The ammonia is then determined with Berthelot's reagent [25].

### 2.6. Nitric Oxide Estimation

NO levels in culture supernatants were assessed via the measurement of residual nitrites by modified Griess's method as previously described by Touil-Boukoffa et al. [26].

### 2.7. Statistical Analyses

The Mann-Whitney *U* test was used for comparisons between groups. ANOVA test was used for multiparametrical comparisons. The results were considered significant when the *P* value was less than 0.05.

## 3. Results

### 3.1. *In Vivo* NO and Urea Production

**Figure 1:**
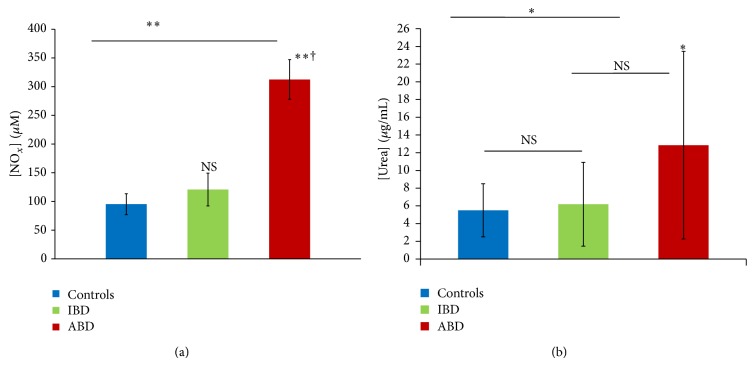
Plasmatic nitric oxide (a) and urea (b) levels in patients with BD (*n* = 36) during active stage (ABD) and inactive stage (IBD) and in control subjects (controls, *n* = 15) (ns: *P* > 0.05; ∗: *P* < 0.05 versus control subjects, ∗∗: *P* < 0.01 versus control subjects, †: ABD versus IBD *P* < 0.05).

In agreement with our previous studies, we noticed that NO production was significantly increased in patients with active BD in comparison to control subjects (*P* < 0.01) while there was no difference between patients during the inactive stage and control subjects (*P* = 0.78) (Figure 1(a)). As observed for nitric oxide, urea production during BD is significantly higher than the control's during the active stage (*P* < 0.05). However, even if patients in the active stage showed an increase of urea production, the difference was not significant (*P* = 0.216) (Figure 1(b)).

### 3.2. *In Vivo* IL-6 and TGF-*β* Production

**Figure 2:**
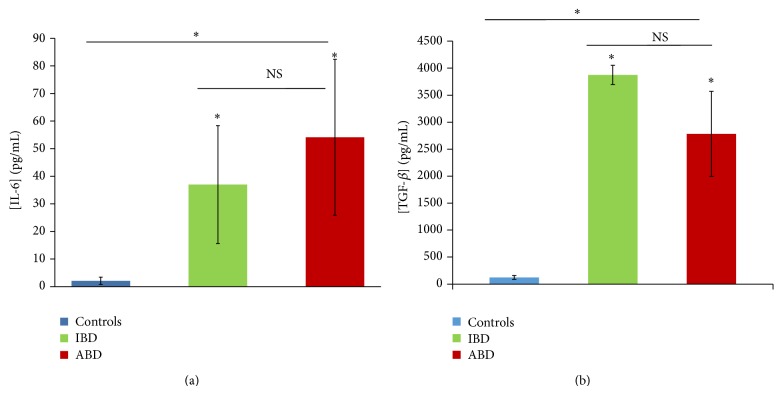
Plasmatic IL-6 (a) and TGF-*β* (b) levels in patients with BD (*n* = 36) during active stage (ABD) and inactive stage (IBD) and in control subjects (controls, *n* = 15) (ns: *P* > 0.05; ∗: *P* < 0.05 versus control subjects).

Several cytokines are implicated in the modulation of NOS and arginase expression and/or activity. In our study, we observed that IL-6 production profile was similar to urea profile* in vivo* (Figure 2(a)). Another ARG modulator, TGF-*β* showed a significant increase during BD with no differences between active and inactive stages (Figure 2(b)).

### 3.3. Cytokine Modulation Effect on Nitric Oxide and Urea Production* Ex Vivo*

#### 3.3.1. IL-6 and TGF-*β* Effect on* Ex Vivo* NO and Urea Production by Controls' PBMC

**Figure 3:**
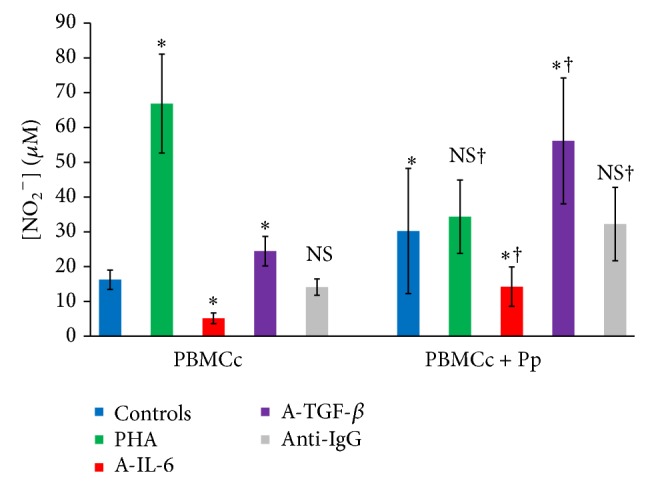
Patients plasma effect on nitric oxide production by cultured controls PBMC (*n* = 23) (ns: *P* > 0.05; ∗: *P* < 0.05 versus control culture without induction; †: *P* < 0.05 versus culture without Pp) (PBMCc: controls' PBMC, control: culture without effectors, Pp: patients' plasma, A-IL-6: anti-IL-6, A-TGF-beta: anti-TGF-*β*, and anti-IgG: isotype control IgG).

In order to determine the implication of the environmental cytokines in NO and urea production, control's PBMC was cultured in RPMI 1640 with FBS (5%) in presence or absence of patients plasma (Pp). Our results showed that Pp increased significantly nitric oxide production* ex vivo*. Mitogenic T cell activation by PHA (10 *μ*g/mL) alone significantly increased NO production but had no significant effect when added with Pp (Figure 3).

Neutralizing IL-6 reduced nitrites levels in both presence and absence of Pp (*P* < 0.05). In contrast, the addition of anti-TGF-*β* increased nitrites levels significantly both in presence and in absence of Pp (*P* < 0.05). However, even if the cells responses had the same profiles, significant differences were observed between the two groups. In fact, TGF-*β* neutralization in Pp treated cultures led to a higher nitric oxide production than in those without Pp (*P* < 0.05).

#### 3.3.2. IL-6 and TGF-*β* Effect on* Ex Vivo* Urea Production by Controls' PBMC

**Figure 4:**
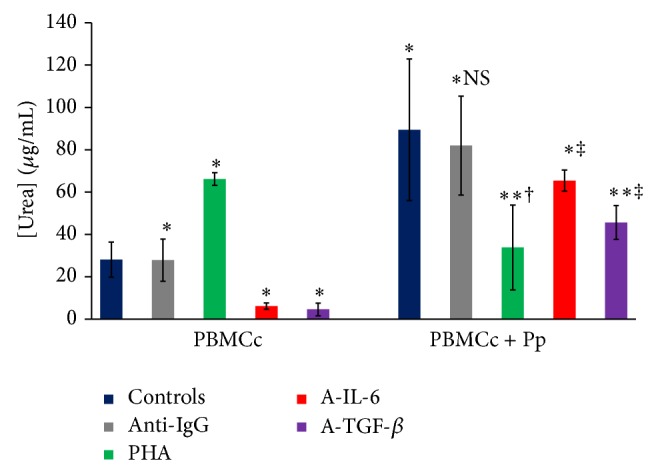
Patients plasma effect on urea production by cultured controls' PBMC (*n* = 23) (ns: *P* > 0.05; ∗: *P* < 0.05 versus control cultures without induction; ∗∗: *P* < 0.01 versus control cultures without induction; †: *P* < 0.05 versus culture without Pp; ‡: *P* < 0.01 versus culture without Pp) (PBMCc: controls' PBMC, Pp: patients' plasma, A-IL-6: anti-IL-6, A-TGF-beta: anti-TGF-*β*, and anti-IgG: isotype control IgG).

As observed for NO production, Pp addition to controls' PBMC cultures induced a significant increase in cultures' supernatants' urea levels (*P* < 0.05). In addition, IL-6 neutralization induced a significant reduction of urea levels in both presence and absence of Pp (*P* < 0.05). However, TGF-*β* neutralization also inhibited the production of urea* ex vivo* (Figure 4). Furthermore, cultures treatment with PHA reduced significantly urea production in the presence of Pp (*P* < 0.05).

#### 3.3.3. IL-6 and TGF-*β* Neutralization Effect on NO and Urea Production by Patients PBMC

**Figure 5:**
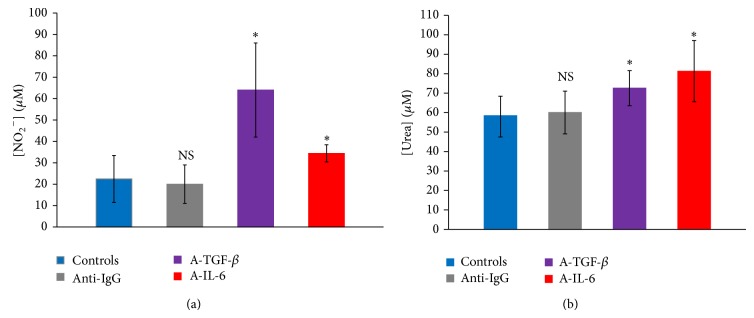
Effect of IL-6 and TGF-*β* neutralization on NO (a) and urea (b) production by patients' PBMC cultures (*n* = 35) in presence of patients' plasma at 20% (controls: cultures without antibodies, A-IL-6: anti-IL-6, A-TGF-beta: anti-TGF-*β*, anti-IgG: isotype control IgG, and ∗: *P* < 0.05 versus control cultures).

When patients' PBMCs were cultured in presence of their plasma, they responded differently from controls' cells to IL-6 neutralization. In fact, the addition of anti-IL-6 (20 *μ*g/mL) to culture medium induced a significant increase in both nitric oxide and urea levels (*P* < 0.05). In the same way, TGF-*β* blockade induced a significant increase in both molecules concentrations in culture supernatants (*P* < 0.05) (Figure 5).

### 3.4. Pharmacological Modulation of Nitric Oxide and Urea Production* Ex Vivo*

#### 3.4.1. Culture Medium Effect on NO and Urea Production

**Figure 6:**
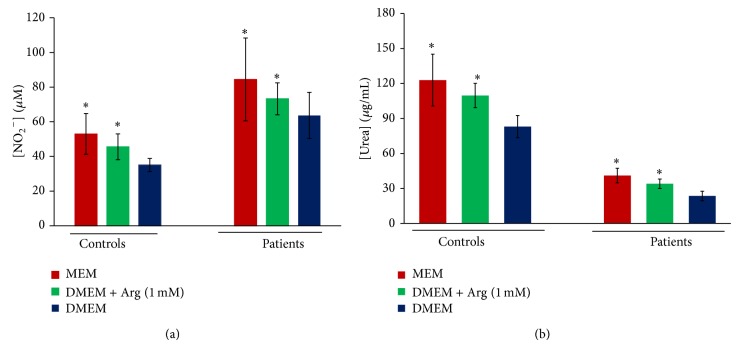
Effect of L-arginine and culture medium on NO (a) and urea (b) production by patients' (*n* = 23) and controls' (*n* = 10) PBMC cultures (controls: control subjects, ∗: significantly different from DMEM with *P* < 0.05).

As NOS and ARG are enzymes, they can be modulated posttranscriptionally by their substrate's availability. In order to investigate the presence of a putative competition between the two enzymes, we cultured PBMC from control subjects and patients with BD in the presence of Pp and in two different culture mediums that differ in their amino-acid composition: MEM, a medium rich in arginine, and DMEM, a medium containing inhibitors for substrate transport and regeneration. Our results showed that nitric oxide as well as urea production was dependent on culture mediums (Figure 6). In fact, both NO and urea levels were higher in MEM than in DMEM (*P* < 0.05). Furthermore, arginine supplementation to DMEM induced a significant increase in NO and urea production in both controls' and patients' cells' supernatants (*P* < 0.05). However, patients PBMC produced higher NO levels than control cells while urea concentrations were significantly lower in patients' cultures' supernatants in comparison to control culture supernatants (*P* < 0.05).

#### 3.4.2. Plasma Effect on NO and Urea Production by Patients PBMC

**Figure 7:**
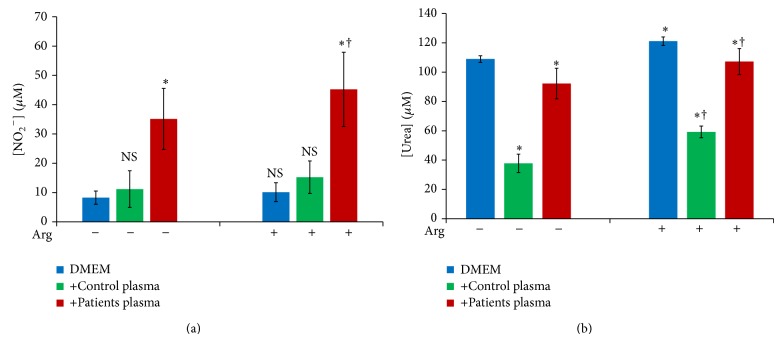
Effect of plasma addition on NO (a) and urea (b) production by patients' PBMC cultures (*n* = 26) in DMEM with or without L-arginine at 1 mM (CM: medium without plasma, ns: *P* > 0.05, ∗: *P* < 0.05 versus CM, and †: *P* < 0.05 versus culture without L-arginine).

In the absence of plasma, addition of arginine to culture medium did not increase nitric oxide concentration in cultures' supernatants (*P* = 0.458) (Figure 7(a)). In contrast, urea levels were increased significantly in presence of L-arginine (Figure 7(b)). Furthermore, in the absence of arginine, patients' PBMC cultures treated with control's plasma showed a slight and not significant increase in nitrites' supernatants' concentrations while urea production was significantly inhibited (*P* < 0.05). In addition, Pp supplementation at 20% increased nitric oxide production while it reduced urea production (*P* < 0.05) (Figures 7(a) and 7(b)). Finally, arginine supplementation to culture medium (DMEM) increased nitric oxide levels in cultures supernatants in presence of patients' plasma but not in presence of controls' plasma. In contrast, arginine addition increased urea production in all cases (*P* < 0.05).

#### 3.4.3. Effect of Metabolic Effectors on Nitric Oxide and Urea Production in Behçet Disease* Ex Vivo*

**Figure 8:**
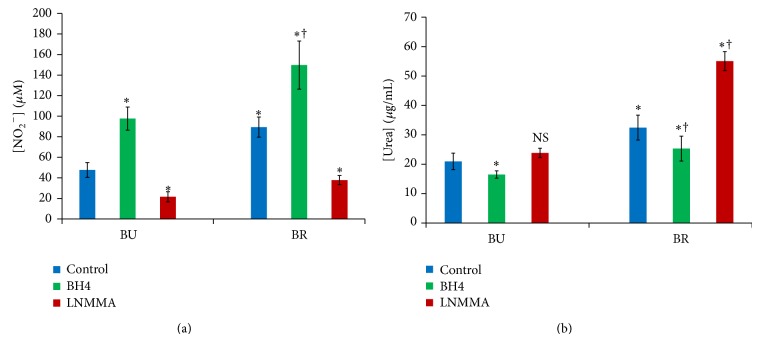
L-NMMA and BH4 effect on nitric oxide (a) and urea (b) concentrations in patients' PBMC cultures supernatants (*n* = 29) in presence of patients' plasma at 20% (BU: uveitis, BR: arthritis, ∗: *P* < 0.05 versus control cultures, and †: *P* < 0.05 between patients categories).

We explored the effect of BH4, a NOS cofactor, and L-NMMA, a NOS substrate competitive inhibitor, on NO and urea production by cultured patients' PBMC in DMEM in presence of Pp. We observed the presence of heterogeneous production profiles depending on the disease clinical expression. In fact, patients with arthritis showed higher production of NO and urea (*P* < 0.05). As expected, in all cases, nitric oxide production was reduced significantly by L-NMMA addition and increased significantly by BH4 supplementation (*P* < 0.05) (Figure 8(a)). However, urea production was differently affected by those treatments. In fact, urea concentrations were reduced significantly after culture treatment with BH4 (*P* < 0.05). In contrast, during arthritis, L-NMMA increased urea production but, during uveitis, urea levels increase was not significant (*P* = 0.19) (Figures 8(a) and 8(b)).

## 4. Discussion

In our study, for the first time, we observed increased urea levels during Behçet disease both* in vivo* and* ex vivo* in comparison to control subjects. As for nitric oxide production, this elevation was only increased during active stage. Moreover, patients' plasma induced increased production of both molecules by either patients or control subjects immune cells. These results suggest that ARG and NOS are overexpressed in Behçet disease and are induced by soluble factors. This is corroborated by IL-6 and TGF-*β* neutralization effect on control subjects' cultured cells* ex vivo*. Behçet disease is characterized by the increased production of several cytokines. IL4, IL-10, and TGF-*β* are potent inducers of arginase 1 and inhibitors of NOS-2, the immune and inducible form of NO synthases [27, 28]. In this study, we showed the presence of high levels of TGF-*β* and we previously showed the production of IL-4 and IL-10 which is especially increased during inactive stages [16–18]. Those cytokines are probably responsible for the increased ARG expression and activity and subsequent urea production. However, NOS-2 is still overexpressed and active. This may be due to the presence of IFN-*γ* and TNF-*α* in patients' plasma. This has been shown during BD with higher production during active stage [12–14]. In our study, patients PBMC showed a different response than the control subjects* ex vivo* in response to their plasma. This may be explained by the presence of secreted cytokines by the cells inducing different response profiles. In fact, IL-6 and TGF-*β* are mainly implicated in the induction of Th17 polarization. This CD4+ relatively new population has been recently implicated in Behçet disease [29, 30] and can be responsible for the modification of cellular responses to cytokine neutralization as it can antagonize the well-defined Th1 responses during BD [12]. Several studies associated the unbalanced cytokines' production during Behçet disease with genetic background. In fact, a strong association was found between IL-10 and IL-23R-IL-12RB2 genes' polymorphisms and the incidence of BD by Genome-wide association study as well as candidate gene approaches [31, 32]. The last gene polymorphism seems to be responsible for higher expression of IL-23 receptor, TNF-*α*, and IL-6 [33]. However, a study on Algerian patients showed a weak association between IL23R-IL12RB2 region SNPs and BD [34]. Other cytokines have shown specific polymorphism associated with BD development or severity. Several studies showed a significant association between TGF-*β* gene polymorphism and BD occurrence [35–37]. This association suggests that TGF-*β* can play a major role during physiopathology of BD. Besides, IL-6 gene polymorphism studies showed the presence of a significant association with BD in Turkish and Korean populations [36, 38]. However, this finding was not confirmed in an Egyptian study [39]. These findings are in agreement with the observed ethnical and geographical differences during BD.

Increased nitric oxide production has been reported in several studies during BD [17, 40, 41]. Nitric oxide is a highly unstable radical in biological environment where it reacts with reactive oxygen species to give highly toxic species as peroxynitrite [42]. Arginine depletion can block nitric oxide synthesis leading to the diminution of its concentrations and the subsequent deleterious effects [43]. Thus, the presence of arginase can exert a protective role during active stage of BD by limiting the lesions caused by nitric oxide production. However, several studies showed that NOS can produce peroxynitrites directly during inflammation in arginine-depleted environment [44, 45]. In this case, the concomitant presence of arginase and NOS worsens the effect of nitric oxide during inflammation. This effect is observed in several diseases with vascular involvement [46]. Further investigations should be conducted to determine the beneficial or deleterious role of arginase during BD.

Metabolic modulation of arginine metabolism showed different production profiles depending on the clinical expression and the studied molecule. Increased levels were observed in MEM cultures supernatants in comparison to DMEM culture supernatants. MEM contains more L-arginine than DMEM. In return, DMEM contains L-lysine, a cationic amino-acid sharing the same cellular transporters with L-arginine (cationic amino-acid transporter: CAT), thus competing with L-arginine in cellular intake [47]. In addition, DMEM contains L-glutamine, an inhibitor of L-arginine regeneration via NO cycle [48]. These two mechanisms seem to play a major role in both NOS and ARG inhibition at the posttranscriptional level. However, the inverted production profiles observed after BH4 and L-NMMA addition to cultures suggest the presence of substrate competition between the two enzymes.

The analysis of arginine metabolism during the different clinical manifestations of BD showed that, when compared to uveitis, arthritis was characterized by a higher production of both NO and urea. This could be due in part to disease activity as almost all patients with arthritis were in the active stage. However, this also can be due to different immune pathways which are responsible for the different clinical manifestations as observed in some studies [49]. The responsiveness in ARG responses to treatment during uveitis can be partially explained by the abundance of arginine in MEM, thus masking the competition between the two enzymes.

## 5. Conclusion

Our results showed* in vivo* increased urea levels in Behçet disease in comparison to control subjects. In addition, it showed the modulation of arginase activity by plasma cytokines notably IL-6 and TGF-*β*. Furthermore, based on substrate availability, we observed the possible presence of a balance between NOS and arginase during the different disease stages. This balance is modulated by the environmental factors that induce or modulate immune responses during Behçet disease. Finally, we observed different metabolism profiles depending on disease activity and/or clinical expression. This last point underlines the presence of different pathophysiological mechanisms implicated in Behçet disease and suggests that arginases, with TGF-*β* and IL-10, are part of the regulatory pathways of immune disorders during Behçet disease.

## Figures and Tables

**Table 1:** Patients' clinical features.

Clinical manifestations	Activity (active/inactive)
Oral ulcerations (100%)	0/36
Genital ulceration (72.22%)	0/26
Uveitis (69.44%)	12/16
Arthritis (41.66%)	13/15
Skin lesions (13.88%)	1/4
Gastrointestinal inflammation (2.78%)	0/1
